# Electrostatic Targeting of Cancer Cell Membrane Models by NA-CATH:ATRA-1-ATRA-1: A Biophysical Perspective

**DOI:** 10.3390/membranes15100303

**Published:** 2025-10-06

**Authors:** Maria C. Klaiss-Luna, Małgorzata Jemioła-Rzemińska, Marcela Manrique-Moreno, Kazimierz Strzałka

**Affiliations:** 1 Chemistry Institute, Faculty of Exact and Natural Sciences, University of Antioquia A.A 1226, Medellin 050010, Colombia; mklaissl@pasteur.fr (M.C.K.-L.); marcela.manrique@udea.edu.co (M.M.-M.); 2 Faculty of Biochemistry, Biophysics and Biotechnology, Jagiellonian University, 30-387 Krakow, Poland; kazimierz.strzalka@uj.edu.pl; 3 Malopolska Centre of Biotechnology, Jagiellonian University, 30-387 Krakow, Poland

**Keywords:** breast cancer, peptide–lipid interactions, biophysical studies, differential scanning calorimetry, Fourier-transform infrared spectroscopy

## Abstract

Breast cancer continues to be the leading cancer diagnosis among women worldwide, affecting populations in both industrialized and developing regions. Given the rising number of diagnosed cases each year, there is an urgent need to explore novel compounds with potential anticancer properties. One group of such candidates includes cationic peptides, which have shown promise due to their unique membrane-targeting mechanisms that are difficult for cancer cells to resist. This study presents an initial biophysical assessment of NA-CATH:ATRA-1-ATRA-1, a synthetic peptide modeled after NA-CATH, originally sourced from the venom of the Chinese cobra (*Naja atra*). The peptide’s interactions with lipid bilayers mimicking cancerous and healthy cell membranes were examined using differential scanning calorimetry and Fourier-transform infrared spectroscopy. Findings revealed a pronounced affinity of NA-CATH:ATRA-1-ATRA-1 for eukaryotic membrane lipids, particularly phosphatidylserine, indicating that its mechanism likely involves electrostatic attraction to negatively charged lipids characteristic of cancer cell membranes. Such biophysical insights are vital for understanding how membrane-active peptides could be harnessed in future cancer therapies.

## 1. Introduction

Biologically active peptides (BAPs) constitute a highly diverse class of small molecules, typically ranging from 5 to 50 amino acids in length, and are found across all domains of life—from microorganisms to mammals [1]. Snake venom is a particularly rich source of BAPs, containing complex mixtures of proteins and peptides known for their varied molecular structures and biological functions [2]. One example is NA-CATH:ATRA1-ATRA1 (NA), a synthetically designed peptide composed of 34 amino acids (KRFKKFFKKLK-NSVKKRAKKFFKKPKVIGVTFPF). This molecule is a modified version of the cathelicidin NA-CATH, originally identified in the venom of the elapid snake *Naja atra*, in which the ATRA1 motif (KRFKKFFKKLK) was incorporated to optimize its properties [3]. NA is a highly cationic (+15) and amphipathic peptide that has demonstrated strong antibacterial activity against *Staphylococcus aureus*, with an EC_50_ value of 0.51 μg/mL (0.09 μM) in both free-living cells and biofilm states [3].

These peptides are primarily recognized for their ability to interact with microbial membranes. However, recent research has expanded their potential applications to include anticancer therapies [4]. A key aspect of their mechanism of action involves electrostatic interactions, which are fundamental to the initial binding between BAPs and target membranes. Specifically, the highly positive charge of peptides like NA promotes selective attraction to negatively charged membranes—such as those of bacteria or cancer cells—while sparing neutral membranes typical of healthy mammalian cells [5].

In cancer cells, the aberrant exposure of phosphatidylserine (PS) on the outer leaflet of the plasma membrane significantly increases the negative surface charge, thereby enhancing the electrostatic affinity for cationic peptides like NA [6,7]. In contrast, normal cell membranes predominantly contain zwitterionic lipids (e.g., phosphatidylcholine and sphingomyelin), resulting in a relatively neutral surface charge that reduces peptide binding [8,9]. This differential electrostatic landscape offers a potential selectivity mechanism that could be exploited for therapeutic purposes.

Once electrostatic attachment occurs, secondary hydrophobic interactions facilitate the insertion of the peptide into the membrane, potentially leading to bilayer disruption through mechanisms such as the carpet model, toroidal pore formation, or barrel-stave channels [10]. This membrane-targeted action bypasses common resistance mechanisms encountered by intracellular chemotherapeutics [11,12], making BAPs especially attractive as anticancer agents. While traditional chemotherapeutics must penetrate cancer cells to be effective, often leading to resistance, BAPs have the advantage of acting from outside the membrane—a mechanism that cancer cells struggle to counteract [13,14].

In previous studies, our group showed that NA exhibits cytotoxic activity against two molecular subtypes of breast cancer—MCF-7 (IC_50_ = 13.4 µM) and MDA-MB-231 (IC_50_ = 6.4 µM) [15]. Nevertheless, little is known about the biophysical basis of NA’s interaction with lipid membranes representative of these cancer cell lines. Understanding the electrostatic component of these interactions is essential for rational drug design and the development of novel peptide-based therapies.

In this study, we investigated the biophysical interactions of NA with model membranes that replicate the lipid composition of MCF-7 and MDA-MB-231 cells, and compared them with a non-tumoral lipid model based on immortalized human keratinocytes (HaCaT). The lipid compositions were previously quantified by Klaiss-Luna et al. [16], enabling the construction of representative membrane systems. Using differential scanning calorimetry (DSC) and Fourier-transform infrared (FT-IR) spectroscopy, we characterized the thermodynamic behavior, structural organization, and molecular dynamics of peptide–lipid interactions. Moreover, the secondary structure of NA in aqueous and lipid environments was evaluated to understand how membrane composition influences its activity. This research highlights the crucial role of electrostatic interactions in the selective activity of NA and provides insight into its potential as an antitumoral agent.

## 2. Materials and Methods

### 2.1. Peptide and Phospholipids

The synthetic peptide NA-CATH:ATRA1-ATRA1 (sequence: KRFKKFFKKLK-NSVKKRFKKFFKKLKVIGVTFPF; Lot. U037QFC180-11/PE6100) was obtained from GenScript (Piscataway Township, NJ, USA). It was produced via solid-phase synthesis and characterized by high-performance liquid chromatography (HPLC) and matrix-assisted laser desorption/ionization time-of-flight (MALDI–TOF) mass spectrometry, confirming a purity of 97.5% and verifying the expected molecular weight.

Phospholipids with 16:0 and mixed 16:0/18:1 acyl chains were sourced from Avanti Polar Lipids (Alabaster, AL, USA), including: 1,2-dipalmitoyl-sn-glycero-3-phosphocholine (DPPC, Lot. 160PC-318), 1-palmitoyl-2-oleoyl-sn-glycero-3-phosphocholine (POPC, Lot. 850457P-500MG-A-211), 1,2-dipalmitoyl-sn-glycero-3-phosphoethanolamine (DPPE, Lot. 160PE-106), 1-palmitoyl-2-oleoyl-sn-glycero-3-phosphoethanolamine (POPE, Lot. 850757P-500MG-B-151), 1,2-dipalmitoyl-sn-glycero-3-phospho-L-serine sodium salt (DPPS, Lot. 840037P-500MG-A-078), 1-palmitoyl-2-oleoyl-sn-glycero-3-phospho-L-serine sodium salt (POPS, Lot. 840034P-25MG-397 A-250), and sphingomyelin extracted from chicken egg (SM, Lot. 860061P-25MG-A-116). Additional reagents, including HEPES, EDTA, NaCl, and other chemicals of analytical grade, were acquired from Sigma-Aldrich (St. Louis, MO, USA).

### 2.2. Phase Transition Measurements by DSC

Phospholipid stock solutions were individually prepared at a concentration of 10 mM. DPPC and SM were dissolved in chloroform, whereas DPPE and DPPS were solubilized in a 70:30 (*v*/*v*) chloroform:methanol mixture. To generate 1 mM multilamellar vesicles (MLVs), appropriate volumes of each lipid stock were combined in glass tubes to match the reported lipid compositions of model membranes. The mixtures corresponded to the following systems: MCF-7 (DPPC/DPPE/SM/DPPS, 47:35:5:13%), MDA-MB-231 (43:33:4:20%), and the non-cancerous HaCaT cell line (DPPC/DPPE/SM, 59:35:6%) [16]. The solvent was removed by a nitrogen stream to form a thin lipid film, which was then hydrated using a buffer solution containing 10 mM HEPES, 500 mM NaCl, and 1 mM EDTA at pH 7.4. MLVs were obtained by subjecting the suspension to alternating vortexing and immersion in hot water (above the lipid phase transition temperature) for 6 min, followed by a brief 1 min sonication.

For peptide interaction studies, NA-CATH:ATRA1-ATRA1 was incorporated during the hydration step at final concentrations of 1, 5, and 10 mol% in the same buffer solution. Calorimetric measurements were carried out as described in [17] using a Nano DSC Series III microcalorimeter equipped with platinum capillary cells (TA Instruments, New Castle, DE, USA). The sample chamber was filled with 400 µL of either the lipid or lipid-peptide suspension, while the reference chamber contained buffer alone. After sealing, the instrument was pressurized to 0.3 MPa, and the system was allowed to equilibrate for 10 min at the starting temperature. Heating scans were conducted at a constant rate of 1 °C/min within the following temperature ranges: 15–60 °C (DPPC), 40–80 °C (DPPE), 35–75 °C (DPPS), 15–50 °C (SM), 15–75 °C (MCF-7 and MDA-MB-231 models), and 15–65 °C (HaCaT model). The primary phase transition temperature (T_m_) and associated enthalpy change (ΔH) were determined using NanoAnalyze version 3.12.0 (TA Instruments, New Castle, DE, USA). Measurement precision was within ±0.1 °C for T_m_ and ±1 kJ·mol^−1^ for ΔH. All thermograms were processed and visualized using Origin Pro 8.0 (OriginLab Corporation, Northampton, MA, USA). DSC measurements were performed in triplicate.

### 2.3. Infrared Spectroscopy Experiments

To prepare the model systems representing breast cancer and non-cancerous membranes, specific phospholipid masses were weighed and combined in glass tubes to obtain 20 mM stock solutions. The lipid compositions used were as follows: MCF-7 (DPPC/DPPE/SM/DPPS at 47:35:5:13%), MDA-MB-231 (43:33:4:20%), and HaCaT (non-tumoral, DPPC/DPPE/SM at 59:35:6%). These mixtures were dissolved in chloroform and stored at −20 °C until use.

For gel-to-liquid crystalline phase transition experiments, supported lipid bilayers (SLBs) at 20 mM were formed directly on the silicon surface of the BioATR II cell integrated with a Tensor II FT-IR spectrometer (Bruker Optics, Ettlingen, Germany). The setup included a mercury cadmium telluride (MCT) detector and was temperature-controlled using a Huber Ministat 125 water bath (Huber, Offenburg, Germany) with a precision of ±0.1 °C. To form the SLBs, 20 µL of lipid stock was deposited on the crystal surface. After solvent evaporation, the resulting lipid film was hydrated for 15 min with 20 µL of buffer (10 mM HEPES, 500 mM NaCl, and 1 mM EDTA, pH 7.4), either in the presence or absence of NA-CATH:ATRA1-ATRA1 at final peptide concentrations of 1, 5, or 10 mol%. Hydration was performed at a temperature above the lipid system’s phase transition.

Infrared spectra were collected across the following temperature ranges: 43–63 °C for MCF-7, 45–65 °C for MDA-MB-231, and 40–60 °C for HaCaT. Each final spectrum represented the average of 120 scans per temperature point, following buffer subtraction, using a spectral resolution of 4 cm^−1^. Lipid phase behavior was assessed by monitoring changes in the symmetric CH_2_ stretching vibrations (2970–2820 cm^−1^), indicative of acyl chain order, and the ester carbonyl stretching region (1725–1740 cm^−1^), which reflects hydration at the lipid interface. Spectral processing was carried out using OPUS 3D software 8.8.4 (Bruker Optics, Ettlingen, Germany), with relevant vibrational regions extracted and baseline-corrected using the Rubberband method at 20% sensitivity. Wavenumber shifts for symmetric stretching bands were tracked as a function of temperature, and the main phase transition temperature (T_m_) was determined from the inflection point of a sigmoidal curve fitted to the data using the Boltzmann function. Curve fitting employed the Levenberg–Marquardt iterative algorithm in Origin Pro 8.0 (OriginLab Corporation, Northampton, MA, USA).

### 2.4. Peptide Conformational Analysis

A peptide solution at a concentration of 3 mg/mL was prepared in buffer (10 mM HEPES, 500 mM NaCl, 1 mM EDTA, pH 7.4), either alone or in combination with small unilamellar vesicles (SUVs). SUVs at 6 mM were assembled based on the lipid compositions representative of the following membrane models: MCF-7 (POPC/POPE/SM/POPS, 47:35:5:13%), MDA-MB-231 (43:33:4:20%), and HaCaT (POPC/POPE/SM, 59:35:6%). Individual lipid components were accurately weighed, dissolved in chloroform, and thoroughly mixed. The solvent was evaporated under a nitrogen stream to form dry lipid films, which were then rehydrated in buffer and subjected to 30 min of sonication (50/60 Hz) at temperatures exceeding the lipid phase transition threshold.

To assess peptide–membrane interactions, the peptide was combined with the SUV suspension to achieve a final concentration of 15 mol%. The mixture was incubated at 37 °C for 5 min prior to spectral acquisition. Infrared spectra of the peptide, both free and in the presence of SUVs, were recorded at 37 °C using a Tensor II FT-IR spectrometer (Bruker Optics, Ettlingen, Germany) equipped with an AquaSpec Transmission Cell. Each final spectrum was the result of averaging 124 consecutive scans. Secondary structure estimation—specifically, proportions of α-helix and β-sheet content—was carried out using the BPROT1 algorithm from the Confocheck FT-IR platform. The method includes internal calibration against a reference database of 43 proteins and provides structural assignments with an estimated deviation of ±4.4%.

### 2.5. Statistical Analysis

Statistical analysis of the changes in T_m_ and ΔH was performed using GraphPad Prism 8.0.1 software (Dotmatics, Bishop’s Stortford, Herts, UK). One-way ANOVA followed by Fisher’s Least Significant Difference (LSD) post hoc test was used for multiple comparisons.

## 3. Results

The biophysical insights into the potential antitumor activity of NA were obtained through a multidisciplinary approach combining several analytical techniques. These methods allowed us to characterize lipid–peptide interactions by examining both structural and dynamic properties of the model membranes and the peptide. This comprehensive analysis provides a deeper understanding of NA’s potential biological effects.

### 3.1. Thermotropic Phase Behavior by DSC

#### 3.1.1. Model Membranes

Model membranes representative of MCF-7 (PC/PE/SM/PS, 47:35:5:13%), MDA-MB-231 (PC/PE/SM/PS, 43:33:4:20%), and HaCaT (PC/PE/SM, 47:27:5%) cell lines were reconstructed based on the phospholipid class composition identified through lipid extraction and high-performance thin-layer chromatography (HPTLC), as previously described [17]. Differential scanning calorimetry (DSC) was used to investigate the thermotropic behavior of these systems and to assess how peptide interactions influence membrane phase properties. The heating profiles obtained from DSC revealed endothermic transitions associated with the conversion of lipids from the lamellar gel phase (L_β_) to the lamellar liquid-crystalline phase (L_α_). From these transitions, key thermodynamic parameters were derived, including the main phase transition temperature (T_m_), which indicates the temperature at which the phase change occurs, and the transition enthalpy (∆H), which reflects the energy absorbed during the process.

**Figure 1:**
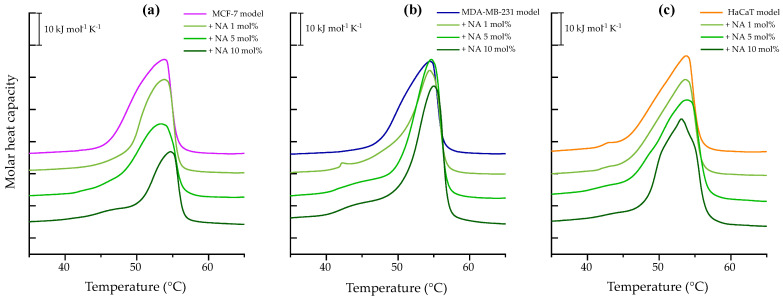
DSC heating scans of 1 mM multilamellar vesicles representative of breast cancer model membranes (**a**) MCF-7 (PC/PE/SM/PS, 47:35:5:13%), (**b**) MDA-MB-231 (PC/PE/SM/PS, 43:33:4:20%), and (**c**) HaCaT (PC/PE/SM, 59:35:6%), in the absence and presence of varying NA concentrations (1, 5, and 10 mol%). The presented thermograms are representative examples from three independent experiments.

Figure 1a illustrates the heating endotherm of breast cancer model membrane MCF-7. The broad peak represents the change from the lipid metastable gel phase to the liquid-crystalline phase with a T_m_ = 53.86 °C and a corresponding transition enthalpy of 38.51 kJ mol^−1^, as reported in Table S1. The presence of the peptide appears to o have a notable effect on peak height reduction as peptide concentration increases. The strongest effect was observed at 10 mol% where NA reduced ΔH to 24.17 kJ mol^−1^ and induced the formation of peptide-poor lipid domains evidenced by a shoulder at 46.19 °C and a shift in the main transition temperature to 54.72 °C.

The thermotropic behavior of breast cancer model membrane MDA-MB-231 is shown in Figure 1b by a broad endothermic peak with a maximum at 54.47 °C and a transition enthalpy ΔH = 41.47 kJ mol^−1^, reported in Table S1. Interaction with NA at 1 mol% induced phase separation of the lipid mixture with an additional peak at 42 °C. At 5 and 10 mol%, the appearance of shoulders suggests the formation of peptide-poor lipid domains. NA also affected the cooperativity of the transitions, as the main transitions became narrower compared to the control. Additionally, an increase in both enthalpy and transition temperature was observed relative to the control peak.

The heating thermogram of HaCaT model membrane, representing human keratinocytes, is presented in Figure 1c. The calorimetric trace revealed a DPPC microdomain at 42.93 °C and a main transition peak at 53.79 °C, with an enthalpy of ΔH = 36.94 kJ mol^−1^. Upon incubation with the peptide, phase separation was observed. It refers to the coexistence of two (or more) distinct lipid populations or domains within the same bilayer, each undergoing its own thermotropic phase transition at a characteristic temperature. This phenomenon can be identified in the thermograms by the presence of multiple peaks or shoulders, rather than a single transition peak. In this case the phase separation was observed at 5 and 10 mol%, with a more pronounced effect at the highest concentration tested. At 10 mol%, NA induced a more cooperative transition and lowered the main transition temperature to 53.10 °C.

**Figure 2:**
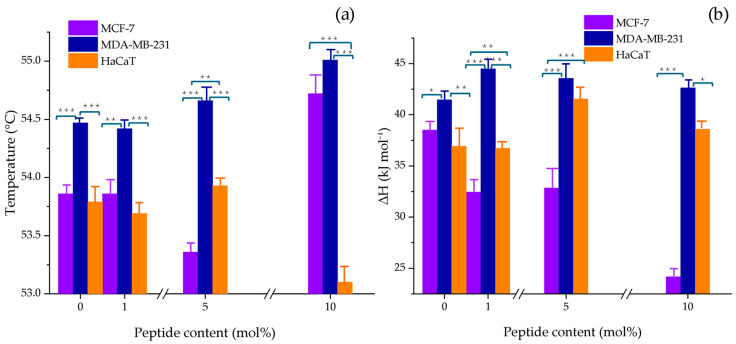
Changes in (**a**) the main phase transition temperature (T_m_) and (**b**) enthalpy (ΔH) of MCF-7 (PC/PE/SM/PS, 47:35:5:13 *w*/*w*), MDA-MB-231 (PC/PE/SM/PS, 43:33:4:20 *w*/*w*), and HaCaT (PC/PE/SM, 59:35:6 *w*/*w*) model membranes as a function of NA content (1, 5, and 10 mol%). The bars represent the mean ± SD from three independent experiments. Statistical significance was assessed at *p* < 0.05. A one-way ANOVA was performed separately for each NA concentration, using the cell line as the factor. When significant differences were found, a Tukey HSD post hoc test was applied to determine pairwise differences between groups; ***, *p* ≤ 0.001; **, *p* ≤ 0.01; *, *p* ≤ 0.05. Detailed values with standard deviations are provided in the Supplementary Table S1.

To comprehensively analyze the effect of the peptide on the thermodynamic parameters, the maximum main transition temperature enthalpy were plotted as a function of peptide concentration across all model membranes, as shown in Figure 2a,b, respectively. The results indicated that the strongest interaction occurred with the breast cancer model MCF-7, showing a change in T_m_ of 0.86 °C and ∆H = 13.82 kJ mol^−1^. No clear trend was observed between the thermodynamic parameters and NA concentration.

#### 3.1.2. Individual Phospholipids

**Figure 3:**
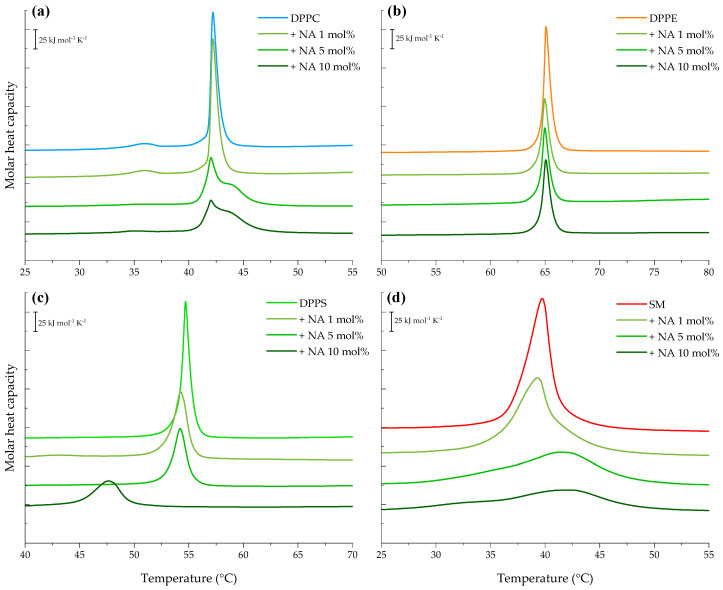
DSC heating endotherms of 1 mM multilamellar vesicles of pure (**a**) DPPC, (**b**) DPPE, (**c**) DPPS, and (**d**) SM in the absence and presence of varying NA concentrations (1, 5, and 10 mol%). The presented thermograms are representative examples from three independent experiments.

Since model membranes are multicomponent lipid systems, we evaluated the interaction between the peptide and individual lipid components—DPPC, DPPE, DPPS, and SM—to better understand and complement the results described above. Starting with the most abundant phospholipid in the constructed models, the thermogram of pure DPPC vesicles, shown in Figure 3a, exhibited the lipid bilayer pretransition corresponding to the change from the gel phase (L_β_) to the ripple phase (P_β_) represented by a small endothermic peak at 35.84 °C with ΔH = 2.84 kJ mol^−1^. The melting process from the ripple to the liquid-crystalline phase (L_α_) was evidenced by the sharp peak at 42.20 °C, indicating a cooperative transition with higher enthalpy ΔH = 32.37 kJ mol^−1^ (see Table S2).

Interaction of NA with DPPC vesicles resulted in the abolishment of the ripple phase at 5 and 10 mol%. Additionally, at these concentrations, the presence of a shoulder was evident, indicating the domain formation. Incubation of the peptide with DPPC liposomes altered the cooperative chain-melting transition, significantly reducing the peak height and enthalpy to ΔH = 24.99 kJ mol^−1^ with minor changes in T_m_.

The heating endotherm of DPPE, the second most abundant lipid in the models, is presented in Figure 3b. Its calorimetric trace revealed a highly cooperative transition by the single sharp peak at 65.04 °C with an enthalpy ΔH = 35.60 kJ mol^−1^. Incubation of NA and DPPE vesicles markedly reduced the characteristic peak height of the DPPE transition at all evaluated concentrations, as reported in Table S2. However, the T_m_ of the pure lipid was not significantly affected.

Furthermore, the heating endotherm obtained for the anionic lipid, DPPS, is illustrated in Figure 3c showing a single sharp transition peak centered at 54.69 °C and a transition enthalpy ΔH = 35.48 kJ mol^−1^. The results highlight that the presence of NA caused significant decrease in the peak enthalpy in a concentration-dependent manner in DPPS liposomes A concomitant reduction in the lipid transition temperature was also observed, reaching 47.6 °C (ΔH = 20.18 kJ mol^−1^) at 10 mol%.

Finally, the SM thermogram is represented in Figure 3d featuring a broad endothermic peak centered at 39.74 °C and a transition enthalpy ΔH = 42.00 kJ mol^−1^, as reported in Table S2. SM seems to be dramatically affected by the presence of NA. Its peak height was reduced by 1.2-fold at 1 mol%, and at the highest concentrations, the phase transition of SM liposomes was almost completely abolished.

**Figure 4:**
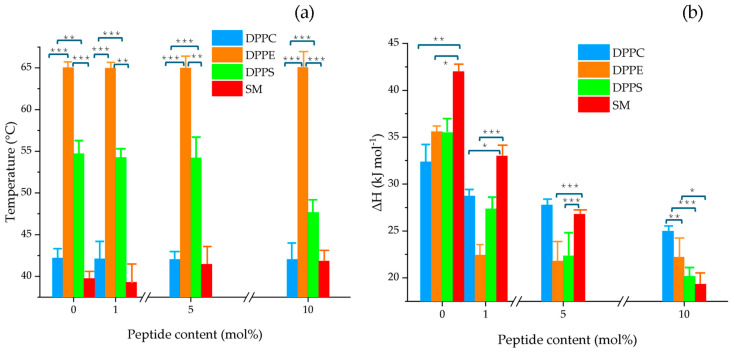
Changes in (**a**) the main phase transition temperature (T_m_) and (**b**) enthalpy (ΔH) of pure DPPC, DPPE, DPPS, and SM multilamellar vesicles as a function of varying NA concentrations (1, 5, and 10 mol%). The bars represent the mean ± SD from three independent experiments. Statistical significance was assessed at *p* < 0.05. A one-way ANOVA was performed separately for each lipid (DPPC, DPPE, DPPS, and SM) to compare the effects of increasing NA concentrations. When significant differences were found, a Tukey HSD post hoc test was applied to determine pairwise differences between groups; ***, *p* ≤ 0.001; **, *p* ≤ 0.01; *, *p* ≤ 0.05. Detailed values with standard deviations are provided in the Supplementary Table S2.

The values of transition temperature and enthalpy as a function of peptide concentration in mol% are shown in Figure 4a,b, respectively. The results indicate that NA did not significantly affect the transition temperature of DPPC and DPPE liposomes; however, it slightly increased the T_m_ of SM vesicles starting from 5 mol%. The strongest effect was observed in DPPS vesicles at 10 mol%, resulting in a net change of T_m_ = 7.04 °C compared to the control.

In terms of enthalpy, a clear decreasing trend was observed with increasing peptide concentration. Figure 4b highlights a pronounced effect in SM and DPPS liposomes, with ΔH values as low as 22.67, and 15.3 kJ mol^−1^, respectively, at 10 mol% NA. These findings suggest stronger interactions of NA with anionic PS and zwitterionic SM lipid systems relative to PC and PE.

### 3.2. Lipid Order and Interfacial Hydration by Infrared Spectroscopy

Membrane structure insights were obtained using infrared spectroscopy in ATR mode to monitor lipid phase transitions and assess the effect of the peptide on this process. This was achieved by analyzing the vibrational bands that offer information about lipid order that refers to the degree of conformational organization of lipid acyl chains in a membrane and the interfacial hydration that refers to the water interactions with the polar headgroups of lipids at the membrane interface. These parameters were followed by the changes in the maximum methylene symmetric stretching (ʋ_s_CH_2_) and the ester carbonyl stretching (ʋ_s_C=O) vibrational modes.

The melting process from the metastable lamellar gel phase to the liquid-crystalline phase is observed in the hydrophobic core of the membrane around 2850–2853 cm^−1^ corresponding to the methylene isomerization from trans to gauche conformations. Meanwhile, interfacial hydration is reflected in the spectral region between 1840 and 1835 cm^−1^ due to the incorporation of water molecules at the carbonyl backbone.

**Figure 5:**
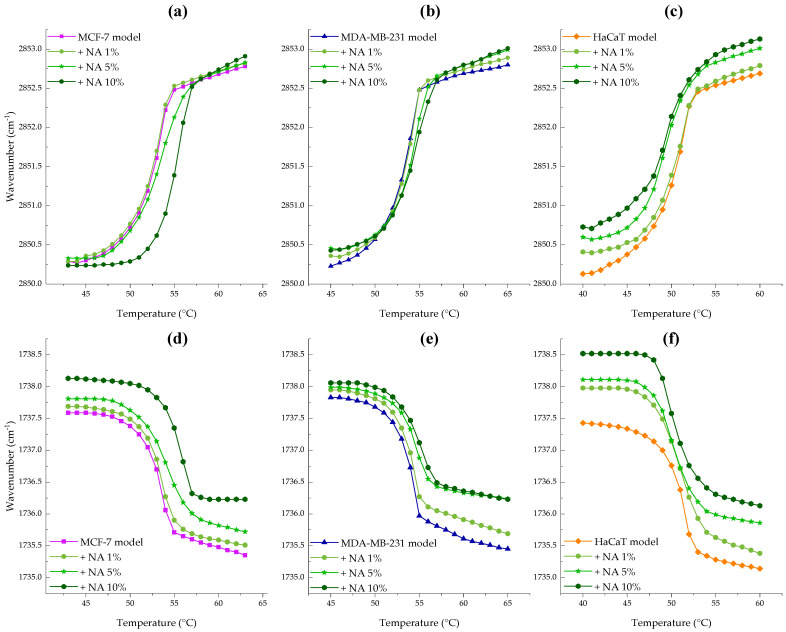
Peptide concentration effect on the maximum symmetric CH_2_ (**a**–**c**) and C=O (**d**–**f**) peak positions of MCF-7 (PC/PE/SM/PS, 47:35:5:13%, ■), MDA-MB-231 ((PC/PE/SM/PS, 43:33:4:20%, ▲) and HaCaT (PC/PE/SM, 59:35:6%, ♦) model membranes as temperature was increased. NA was evaluated at 1 (●), 5 (★), and 10 (⬢) mol%.

Figure 5a–c illustrate the effect of the peptide on the maximum symmetric stretching vibration of CH_2_. For the breast cancer model membranes NA significantly shifted the initial T_m_ of the MCF-7 model to higher temperatures as seen in Figure 5a and data in Table S3. Incubation with the peptide reduced the fluidity of the MCF-7 bilayer resulting in a T_m_ = 55.1 °C (ΔT_m_ = 2.5 °C) with a notable effect on wavenumbers associated with liquid-crystalline phase. This finding suggests that NA-CATH-ATRA1-ATRA1 binds strongly via electrostatic interaction with the negatively charged headgroups, relieving charge repulsion and promoting tighter packing of fatty acid chains, particularly in the gel phase.

The interaction between MDA-MB-231 membranes and NA-CATH:ATRA1-ATRA1, shown in Figure 5b, revealed an increase in wavenumbers for both L_β_ and L_α_ phases as peptide concentration increased, along with a transition temperature shift of ΔT_m_ = 1.2 °C to higher values, indicating increased membrane rigidity. In contrast, the non-tumoral HaCaT model membrane exhibited the opposite trend. Figure 5c shows an increase in wavenumbers for both L_β_ and L_α_ phases with increasing peptide concentration, accompanied by T_m_ shift to lower temperatures (see Table S3). This suggests a fluidizing effect of NA on the HaCaT membrane, which was most pronounced at 10 mol%, resulting in a net ΔT_m_ = 0.8 °C.

Figure 5d–f represents the peptide effect on the maximum symmetric stretching vibration of the C=O group. The interaction between NA and MCF-7 supported lipid bilayers (SLBs), shown in Figure 5d, revealed a strong exclusion of water molecules, as indicated by increasing wavenumbers with rising peptide concentration—an effect more pronounced in the evidenced in the Lα phase. Similarly, the MDA-MB-231 model membrane (Figure 5e) showed a dehydration effect of the carbonyl group upon peptide binding. Finally, incubation of HaCaT SLBs with increasing concentrations of NA (Figure 5f) also resulted in a shift toward higher wavenumbers. These results indicate an overall weakening of hydrogen bonding, suggesting that the peptide binds to and displaces water molecules from the lipid interface.

### 3.3. Secondary Structure Analysis of the Peptide

The secondary structure of NA in solution was studied using infrared spectroscopy. The BPROT1 internal calibration of the peptide spectra indicated that NA is unordered in aqueous buffer and in the presence of neutral POPC small unilamellar vesicles. However, it adopted a slightly α-helix conformation in the presence of multicomponent lipid vesicles. The conformational change was negligible with the HaCaT model membrane but more pronounced with the tumoral model membranes, particularly in the presence of MCF-7 vesicles. The most significant α-helical content was observed in the interaction with anionic POPS vesicles showing 62% α-helix adoption (Table 1).

**Figure 6:**
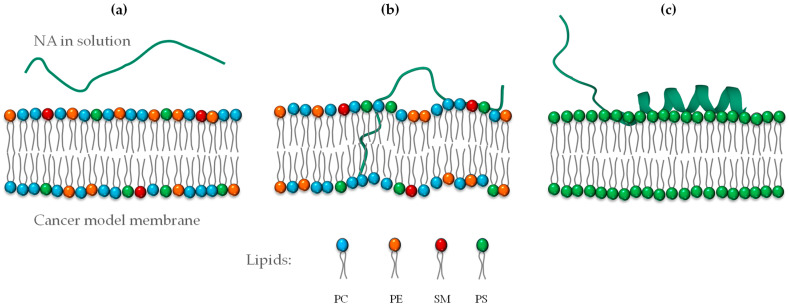
Representation of the secondary structure of NA depending on the lipid composition environment: (**a**) NA in buffer, (**b**) interaction with a cancer model membrane, and (**c**) PS membrane (negatively charged).

These results suggest that NA is an environment-sensitive peptide responding to negatively charged and hydrophobic environments that stabilize its conformational structure. An illustration of the secondary structure of NA is represented in Figure 6.

## 4. Discussion

BAPs have gained attention as promising therapeutic candidates for the treatment of a wide range of human diseases. In this study, we focused on the synthetic peptide NA, with the amino acid sequence KRFKKFFKKLKNSVKKRFKKFFKKLKVIGVTFPF. This peptide is a modified version of the host defense peptide NA-CATH, originally derived from the Chinese cobra *Naja atra*. Specifically, NA was engineered by replacing alanine with phenylalanine at position 18 and substituting proline with leucine at position 25. These modifications were intended to enhance the peptide’s hydrophobicity and improve its antimicrobial efficacy, particularly against *Staphylococcus aureus*, as previously demonstrated [3].

Dean et al. observed that NA exhibited superior antimicrobial and antibiofilm activities compared to the parental peptide, likely due to structural features that favor interactions with anionic membrane environments [3]. Given that many BAPs display dual functionality—namely, antimicrobial and antitumoral properties [18]—our research group has explored the potential antitumoral effects of NA. Previous studies showed that this peptide significantly reduced cell viability in breast cancer cell lines MCF-7 and MDA-MB-231, suggesting a promising therapeutic effect [19]. However, its precise mechanism of action remains unclear. The present study aimed to advance our understanding of the molecular interactions between NA and lipid membranes, using model systems representative of biological membranes. We employed biophysical methods to investigate the peptide’s effects at the membrane level under controlled conditions, with the goal of elucidating its potential antitumoral mechanism.

This section discusses the findings in light of both the physicochemical characteristics of the peptide and the lipid composition of the model membranes used. Regarding the peptide’s properties, its net positive charge is widely recognized as a critical factor influencing biological activity [20]. The interaction between cationic peptides and negatively charged membranes is primarily driven by electrostatic forces. The overall charge of a peptide plays a key role in balancing antimicrobial effectiveness and minimizing undesired hemolytic effects [21].

In a systematic investigation, Dathe and colleagues examined how altering the charge within the magainin peptide family affected both membrane activity and selectivity. Their results demonstrated that increasing the number of positive charges enhanced electrostatic interactions with target membranes, improving antimicrobial performance while reducing hemolytic activity, thus increasing specificity [22]. Similarly, Jiang et al. evaluated how variations in net charge and the distribution of positively charged residues along the hydrophilic face of α-helical peptides influenced their biological function. Their findings revealed that a net charge below +4 led to complete loss of antimicrobial and hemolytic activity in V13K analogs, whereas gradually increasing the charge from +4 to +8 significantly improved their antimicrobial potency [23].

The composition of lipid membranes plays a critical role in modulating the activity of BAPs. In this study, the inclusion of PS in cancer-mimicking membrane models reflects the characteristic negative surface charge observed in tumor cells. This phenomenon is attributed to the abnormal externalization of PS to the outer leaflet of the plasma membrane, along with elevated levels of other anionic components such as heparan sulfates, sialylated gangliosides, and O-glycosylated mucins [24,25]. Using flow cytometry, Iwasaki et al. established a correlation between PS expression levels and the susceptibility of cancer cell lines to antimicrobial peptides. By testing four D-enantiomeric analogs of beetle defensins (peptides A–D), they demonstrated selective cytotoxicity linked to PS abundance, providing direct evidence for the role of membrane surface charge in mediating peptide-induced anticancer effects [26].

DSC results in this study revealed notable alterations in the thermotropic behavior of cancer cell model membranes upon exposure to NA, likely driven by strong electrostatic interactions between the highly cationic peptide (net charge +15) and negatively charged vesicles. The interaction profile differed between MCF-7 and MDA-MB-231 models, as reflected in the thermodynamic parameters detailed in Section 3.1.1. In MCF-7 mimic vesicles, the data suggest the formation of lipid domains with low peptide content, possibly due to partial insertion or surface binding that disrupts the gel phase and promotes the liquid-crystalline state. In contrast, at 10 mol% NA, an increase in T_m_ was observed, indicating gel phase stabilization—likely through enhanced interactions with lipid headgroups or acyl chains, leading to tighter packing and reduced membrane fluidity. This may also result from peptide aggregation or ordered structure formation within the bilayer.

In the MDA-MB-231 model, NA consistently increased T_m_ at both 5 and 10 mol%, suggesting gel phase stabilization. This effect may stem from reduced electrostatic repulsion among PS headgroups, which are more abundant in this system, allowing closer lipid packing and stronger van der Waals interactions [27].

Conversely, in the non-tumoral HaCaT model, NA exhibited a membrane-fluidizing effect without significant changes in enthalpy, which points to a superficial interaction primarily involving the polar headgroups. Altogether, these findings indicate that NA modifies the physical state of membranes in a manner that depends not only on peptide concentration but also on the specific lipid composition of the target membrane.

Due to the broad and overlapping endothermic transitions observed in multicomponent lipid systems, we extended our analysis to assess NA’s interaction with individual lipid vesicles composed of PC, PE, PS, and SM. These experiments aimed to clarify the peptide’s specific effects on distinct lipid headgroups. Our findings show that NA exhibits a marked affinity for phosphatidylcholine, particularly at higher peptide concentrations, leading to significant disruptions in the vesicle’s physical behavior. Previous studies have shown that cationic molecules frequently interact with the PC headgroup due to its surface charge characteristics. Such interactions may alter the dipole orientation between the trimethylammonium group [N(CH_3_)_3_^+^] and adjacent water molecules, thereby modifying the spatial configuration of the phosphate group [28]. This mechanistic insight is consistent with our observation of the disappearance of the ripple phase in the PC thermogram following NA exposure. Given that phosphatidylcholine is the most abundant zwitterionic phospholipid in mammalian membranes [29], such disruptions might suggest a potential for cytotoxicity. However, earlier studies have reported low cytotoxic effects of NA in host cells, supporting its selective activity [3,30,31].

The interaction with DPPE vesicles appears to involve peptide-induced lipid clustering and initial hydrophobic associations, similar to the mechanism proposed by Willumeit et al. in their work with the NK-2 peptide [32]. These findings contribute to a better understanding of the role of individual lipid components in modulating peptide–membrane interactions.

Although all phospholipid types appeared to be influenced by the presence of the peptide, analysis of the thermodynamic parameters (Table S2) highlights a notably selective impact on PS and SM systems. Our data suggest that NA exhibits a pronounced electrostatic affinity for PS and. this interaction likely promotes microdomain formation and phase separation, possibly due to peptide intercalation between acyl chains, which disrupts lipid organization by weakening van der Waals forces.

In the case of SM, the disappearance of its phase transition in the presence of NA indicates destabilization of membrane structure, likely mediated by specific interactions between the peptide and the sphingosine backbone, involving both intra- and intermolecular hydrogen bonding at the bilayer interface. These observations are consistent with findings from FTIR-ATR spectroscopy, which align with the calorimetric data. The interaction between NA and MCF-7 lipid models leads to dehydration of the interface and reduced electrostatic repulsion, ultimately increasing lipid packing [33].

In contrast, the presence of NA in MDA-MB-231 membranes appears to favor the liquid-crystalline state, generating a more rigid and dehydrated system, which might facilitate peptide insertion. This is of particular relevant given that biological membranes are predominantly in the fluid phase under physiological conditions, which may enhance NA’s antitumor potential [34,35].

Moreover, experiments involving HaCaT supported lipid bilayers revealed a peptide-induced increase in membrane fluidity, likely related to water displacement from the interfacial region—a phenomenon that may differ depending on the lipid environment [36]. The link between membrane-active peptide structure and biological activity has been well documented. These peptides often undergo conformational transitions upon membrane contact, typically shifting from disordered to helical forms. Cathelicidins, the peptide class to which NA belongs, are known to adopt amphipathic α-helical structures when interacting with lipid bilayers. LL-37, the human cathelicidin, is among the most extensively characterized examples [37,38,39]. Factors influencing this structural transition include peptide properties such as sequence, net charge, hydrophobicity, and amphipathicity, as well as the lipid composition of the target membrane.

In this work, we employed FT-IR spectroscopy to examine the secondary structure of NA in both aqueous and lipid environments. The technique is well recognized for the determination of protein secondary structure in peptides and proteins [40,41,42]. The results demonstrated that a defined helical structure emerged only in the presence of negatively charged lipids, particularly POPS vesicles, indicating that electrostatic interactions are crucial for conformational ordering. These findings support the observations by Dean et al., who reported increased helicity of NA in 50% TFE and SDS environments using circular dichroism [3]. Interestingly, NA did not undergo the same structural transition in MCF-7 or MDA-MB-231 membrane models, which contain lower levels of PS, suggesting that the peptide may exert its biological function without requiring a fully ordered structure.

In summary, our findings suggest a preferential localization and interaction of NA with cancer cell membranes. In MCF-7 and MDA-MB-231 models, initial electrostatic binding to PS appears to mitigate charge repulsion and may induce partial helical folding, promoting peptide insertion, particularly in the more disordered MDA-MB-231 membranes. In contrast, NA remains at the surface of HaCaT membranes, interacting mainly with the polar headgroups without penetrating the bilayer, representing an energetically less favorable scenario for conformational change

These insights complement previous biological findings and underscore the therapeutic potential of NA-CATH-ATRA1-ATRA1, although further studies are necessary to fully delineate its mechanism of action.

## 5. Conclusions

This biophysical investigation provides valuable insights into the molecular mechanisms underlying the potential antitumor effects of NA-CATH-ATRA1-ATRA1, previously demonstrated by our research group in breast cancer cell lines. Our findings emphasize the critical role of both the peptide’s cationic and hydrophobic properties as well as the specific lipid composition of the target membrane. Notably, the MCF-7 tumor model membrane exhibited a more pronounced response to peptide binding compared to the MDA-MB-231 model, likely due to stronger electrostatic and hydrophobic interactions that facilitate peptide insertion into the bilayer’s hydrophobic core. In contrast, in the non-tumoral HaCaT membrane model, the peptide appears to remain associated with the membrane surface. Additionally, secondary structure analysis revealed that NA-CATH-ATRA1-ATRA1 retains its biological activity without adopting a helical conformation when interacting with the tumor-derived membranes.

## Figures and Tables

**Table 1:** Conformational analysis of NA in aqueous and hydrophobic lipid environments at 37 °C. The helical increase percentage was calculated with respect to buffer and has a deviation of ±4.4%.

	SUVs	α-Helical Content (%)
	Hepes *	-
	POPC	-
NA-CATH-ATRA1-ATRA1 +	POPS	62.2
	MCF-7	4.2
	MDA-MB-231	1.2
	HaCaT	0.3

* Buffer described in Section 2.4.

## Data Availability

The original data presented in the study about the lipid composition of model membranes are openly available at [10.3390/ijms242216226]. The original contributions presented in the study are included in the article/supplementary material; further inquiries can be directed to the corresponding authors.

## Supplementary Materials

The following supporting information can be downloaded at: https://www.mdpi.com/article/10.3390/membranes15100303/s1, Table S1. Main phase transition temperature (T_m_), enthalpy (∆H), and entropy (∆S) from DSC heating endotherms of breast cancer MCF-7, MDA-MB-231, and human keratinocytes HaCaT multilamellar liposomes at varying NA concentrations (1, 5 and 10 mol%), Table S2. Main phase transition temperature (T_m_), enthalpy (∆H), and entropy (∆S) from DSC heating endotherms of pure DPPC, DPPE, DPPS, and SM multilamellar liposomes at varying NA concentrations (1, 5, and 10 mol%), Table S3. Main phase transition temperature (T_m_) of the MCF-7, MDA-MB-231, and HaCaT model membranes in the presence and absence of NA at 1, 5, 10 mol% obtained using infrared spectroscopy.
