# Supplementary material for: Electrostatic Targeting of Cancer Cell Membrane Models by NA-CATH:ATRA-1-ATRA-1: A Biophysical Perspective
Source: Membranes (Basel). 2025 Oct 6;15(10):303. doi: 10.3390/membranes15100303 (PMC12566102; doi:10.3390/membranes15100303)
Supplement: Supplementary file 1 [file membranes-15-00303-s001.zip › Table S1.pdf]

Table S1. Main phase transition temperature (T_m), enthalpy (ΔH), and entropy (ΔS) from DSC heating endotherms of breast cancer MCF-7, MDA-MB-231, and human keratinocytes HaCaT multilamellar liposomes at varying NA concentrations (1, 5 and 10 mol %). The standard deviation was calculated based on calorimetric profiles obtained from three independent measurements.

	Heating		
	**T_m [°C]**	**ΔH [kJ mol^{-1}]**	**ΔS [kJ mol^{-1} K^{-1}]**
**MCF-7**	53.86±0.18	38.51±1.25	0.12
+ 1 mol% NA	53.86±0.30	32.46±1.45	0.10
+ 5 mol% NA	53.36±0.18	32.85±1.20	0.10
+ 10 mol% NA	54.72±0.15	24.17±1.27	0.07
**MDA-MB-231**	54.47±0.31	41.47±1.37	0.13
+ 1 mol% NA	54.42±0.19	44.49±1.19	0.14
+ 5 mol% NA	54.66±0.28	43.57±1.17	0.13
+ 10 mol% NA	55.01±0.31	42.63±1.53	0.13
**HaCaT**	42.93+0.23 and 53.79±0.21	36.94±1.04	0.11
+ 1 mol% NA	53.69±0.21	36.74±1.34	0.11
+ 5 mol% NA	53.93±0.16	41.54±1.44	0.13
+ 10 mol% NA	53.10±0.25	38.59±1.50	0.12
