# Supplementary material for: Electrostatic Targeting of Cancer Cell Membrane Models by NA-CATH:ATRA-1-ATRA-1: A Biophysical Perspective
Source: Membranes (Basel). 2025 Oct 6;15(10):303. doi: 10.3390/membranes15100303 (PMC12566102; doi:10.3390/membranes15100303)
Supplement: Supplementary file 1 [file membranes-15-00303-s001.zip › Table S2.pdf]

Table S2. Main phase transition temperature (T_m), enthalpy (ΔH), and entropy (ΔS) from DSC heating endotherms of pure DPPC, DPPE, DPPS, and SM multilamellar liposomes at varying NA concentrations (1, 5, and 10 mol %). The standard deviation was calculated based on calorimetric profiles obtained from three independent measurements.

	**Heating**		
	**T_m [°C]**	**ΔH [kJ mol^{-1}]**	**ΔS [kJ mol^{-1} K^{-1}]**
**DPPC**	42.20±0.12	32.37±0.85	0.10
+ 1 mol% NA	42.10+0.20	28.74±1.36	0.09
+ 5 mol% NA	42.02+0.19	27.76±1.50	0.09
+ 10 mol% NA	42.03+0.25	24.99±1.34	0.08
**DPPE**	65.04±0.31	35.60±0.30	0.11
+ 1 mol% NA	64.97±0.23	22.44±1.40	0.07
+ 5 mol% NA	64.98+0.22	21.81+1.01	0.06
+ 10 mol% NA	65.06+0.33	22.22±0.52	0.07
**DPPS**	54.69±0.32	35.48±1.51	0.11
+ 1 mol% NA	54.25±0.27	27.36±1.52	0.08
+ 5 mol% NA	54.21±0.19	22.35±0.81	0.07
+ 10 mol% NA	47.65±0.23	20.18±1.05	0.06
**SM**	39.73±0.21	42.00±1.64	0.13
+ 1 mol% NA	39.29±0.16	33.01±0.69	0.11
+ 5 mol% NA	41.46±0.18	26.79±0.70	0.09
+ 10 mol% NA	41.84±0.17	19.33±0.94	0.06
