# Supplementary material for: Electrostatic Targeting of Cancer Cell Membrane Models by NA-CATH:ATRA-1-ATRA-1: A Biophysical Perspective
Source: Membranes (Basel). 2025 Oct 6;15(10):303. doi: 10.3390/membranes15100303 (PMC12566102; doi:10.3390/membranes15100303)
Supplement: Supplementary file 1 [file membranes-15-00303-s001.zip › Table S3.pdf]

Table S3. Conformational analysis of NA in aqueous and hydrophobic lipid environments at 37 °C. The helical increase percentage was calculated with respect to buffer and has a deviation of ± 4.4%.

		SUVs	α-helical content (%)
NA-CATH-ATRA1-ATRA1	+	Hepes*	-
		POPC	-
		POPS	62.2
		MCF-7	4.2
		MDA-MB-231	1.2
		HaCaT	0.3

*Buffer described in Section 2.4.
